# Supplementary figures and images for: A pilot study of a single intermittent arm cycling exercise programme on people affected by Facioscapulohumeral dystrophy (FSHD)
Source: PLoS One. 2022 Jun 24;17(6):e0268990. doi: 10.1371/journal.pone.0268990 (PMC9231774; doi:10.1371/journal.pone.0268990)

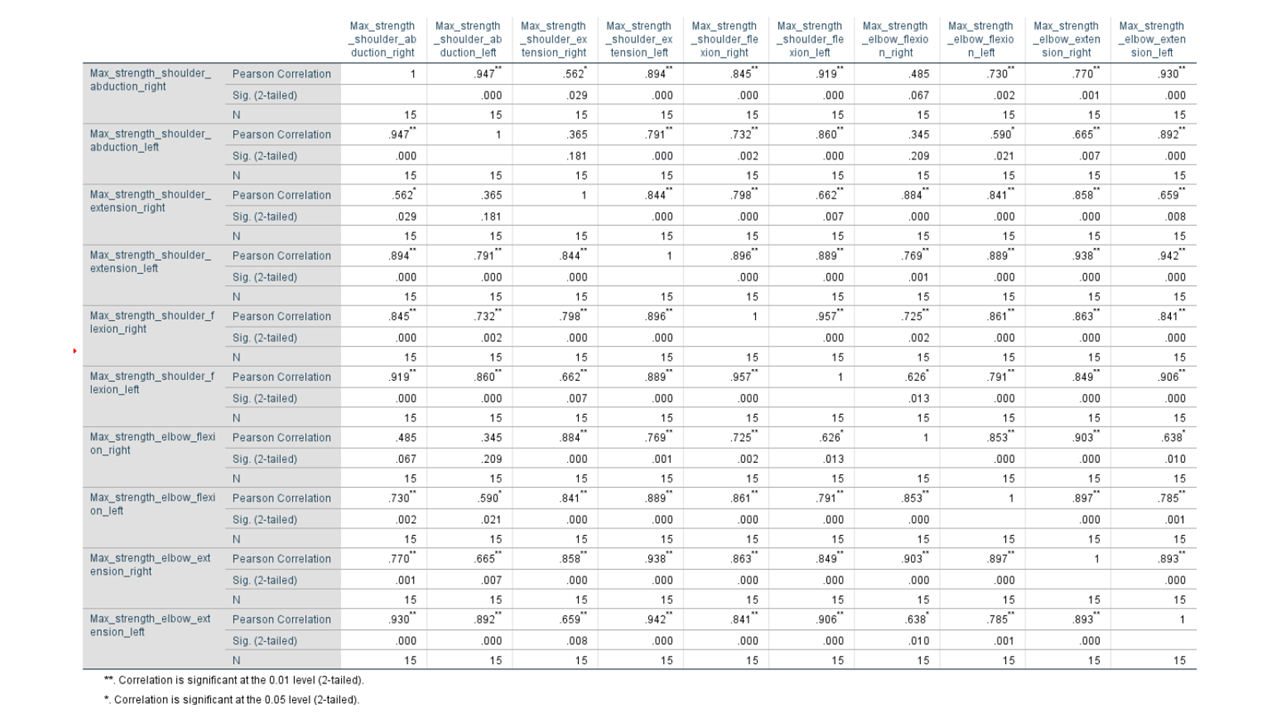

Supplement: S1 Fig — Right maximum shoulder flexion strength was found to be highly correlated (range 0.725 to 0.957) with all other measures of shoulder and elbow strength. Selection of strength in this plane was also supported by the fact that people affected by FSHD often lose functional overhead movement in this plane. (TIF) [file pone.0268990.s001.tif]
