# Supplementary material for: A pilot study of a single intermittent arm cycling exercise programme on people affected by Facioscapulohumeral dystrophy (FSHD)
Source: PLoS One. 2022 Jun 24;17(6):e0268990. doi: 10.1371/journal.pone.0268990 (PMC9231774; doi:10.1371/journal.pone.0268990)
Supplement: S2 File — (PDF) [file pone.0268990.s003.pdf]

***Title:* Arm cycling in Facioscapulohumeral Dystrophy (FSHD) patients**

Short title: Arm cycling in FSHD

PROTOCOL

Version 1.0, 05/JULY/2016

Chief Investigator: **Dr Tracey Willis**
Consultant Neurologist
The Robert Jones and Agnes Hunt Orthopaedic Hospital NHS Foundation Trust

Other Investigators: **Dr Richa Kulshrestha**
Consultant Neurodisability
The Robert Jones and Agnes Hunt Orthopaedic Hospital NHS Foundation Trust

Mr Nicholas Emery
Specialist Neuromuscular Physiotherapist
The Robert Jones and Agnes Hunt Orthopaedic Hospital NHS Foundation Trust

Dr Marco Arkesteijn
Sport and Exercise Research Scientist
Aberystwyth University

Study coordinator **Dr Sarah Turner**
Research Project Manager
The Robert Jones and Agnes Hunt Orthopaedic Hospital NHS Foundation Trust

Sponsor **The Robert Jones and Agnes Hunt Orthopaedic Hospital NHS Foundation Trust**

Funder **Orthopaedic Institute Ltd, Oswestry**

BACKGROUND

FSHD sufferers live a long life with disability. Symptoms may develop in early childhood and weakness usually noticeable in the teenage years with 95% of affected individuals manifesting disease by age 20 years. The disorder impacts on the upper extremity and torso, impacting negatively on the muscle mass, shoulder mobility and functional tasks[1]. Consequently chronic disuse of the shoulder negatively impacts independence of sufferers, prospects of employment and staying at work. Secondary features of chronic pain and fatigue also impact quality of life[1,2]. The disease progression is slow, but highly variable and does not affect life expectancy[3]. Cost of illness analysis per FSHD patient has been estimated to be around £7000 per annum[4]. At present there is no known cure and knowledge regarding the mechanisms underpinning FSHD is not sufficient to halt the progression of the disease via pharmacological interventions or gene therapy. Surgical interventions are used to improve scapular stabilisation[5] but long term effect on disease progression is limited[5,6].

Several studies have shown that exercise with moderate weights or resistance is not detrimental to sufferers with FSHD[7,8]. More recently, a study showed that consistent aerobic training in sufferers with FSHD not only improves cardiovascular fitness but also improves strength[9]. Two systematic reviews published in 2013 identified that moderate intensity exercise is generally not harmful in muscular dystrophy sufferers[10,11]. However detrimental effects are possible, dependent on the protocol and subject[10,11]. Both systematic reviews concluded that there is insufficient evidence regarding its effectiveness. In addition, these findings mainly apply to lower extremity exercise and only one study focussed specifically on FSHD sufferers. Assisted arm cycling (where the crank is moving itself) has been used successfully in young Duchenne Muscular Dystrophy sufferers[12]. In a case study of a severely affected FSHD patient (including loss of mobility), assisted arm cycling was employed as part of an exercise intervention[13]. The passive nature of this task could potentially explain the lack of improvement in the majority of functional assessments. Thus the feasibility of maintaining arm cycling for exercise purposes at an intensity that will induce muscular adaptions is currently unknown.

We believe that arm cycling has the potential to be effective in maintaining or improving shoulder muscle functioning. However, there is no evidence for its safety or effectiveness in the upper extremity. Our aim is to establish the ability of FSHD sufferers to perform arm cycling (stage 1) to underpin the future design of an arm exercise trial (stage 2). Our hypothesis is that the limited range of shoulder movement and muscle weakness should not impact the ability of FSHD sufferers to perform arm cycling.

STUDY OBJECTIVES

PRIMARY OBJECTIVE

The main objective of this stage of the study is to determine whether FSHD sufferers are physically capable of performing arm cycling.

SECONDARY OBJECTIVES

The secondary objective of this stage of the study is to determine what duration, resistance and cadence (speed) of arm cycling each participant can achieve.

TRIAL DESIGN

This is a pilot study to determine the feasibility of arm cycling as an exercise intervention in FSHD sufferers and to inform the design of a larger trial of an arm cycling exercise intervention.

PARTICIPANT SELECTION AND ENROLMENT

Potential participants will initially be identified from the medical records kept for FSHD patients at the RJAH Orthopaedic Hospital, by members of the neuromuscular clinical care team. Medical notes will be reviewed for patients that seem to be eligible for the study, then the invitation letters sent to their home address. They will also be sent a reply slip and a pre-paid envelope, to inform the study team whether they are interested (or not) in taking part in the study.

Once potential participants have indicated that they are interested in taking part in the study, the CI or another suitably qualified member of the research team will determine whether they are eligible via a telephone call, as per the inclusion/exclusion criteria below. Should we need to recruit participants via the FSHD register and/or FSH-MD charity, potential participants will only be approached (by telephone) after they have registered their interest in taking part in the study. Confirmation of molecular genetics result from local neurologist/ GP/ molecular genetics lab will be organised for potential participants prior to the visit.

INCLUSION CRITERIA

Patients will be deemed eligible to take part in the study if they full fill the following inclusion criteria:

1. Aged 18-60 years

2. Genetically confirmed diagnosis of FSHD

3. Willing to attend the assessment session

4. Able to understand the participant information sheet and provide written informed consent

EXCLUSION CRITERIA

Patient will be deemed ineligible for the study if they fulfil any of the following exclusion criteria:

1. Aged <18 years or >60 years

2. Co-morbidity that would affect their ability to perform arm cycling

3. Unwilling to complete the assessments of the study

4. Unable to understand the participant information sheet and provide written informed consent.

WRITTEN INFORMED CONSENT

Written informed consent will be obtained from eligible participants upon their arrival at the Robert jones and Agnes Hunt Orthopaedic Hospital for the study assessment session. The participant will have the opportunity to discuss the study and ask any questions they may have before consent is obtained by the CI or an appropriately qualified member of the research team. The original consent form will be stored in the trial master file, a copy given to the participant for their records and a copy will be added to their medical notes (where available).

Following consent, the participants will be allocated a trial specific ID number. This ID number will be used on all subsequent trial paperwork relating to the participant.

STUDY PROCEDURES

Participants will be asked to attend **one** assessment visit at the Robert Jones and Agnes Hunt Orthopaedic Hospital NHS Foundation Trust. Participants will undergo an arm cycling exercise session. Prior to exercise patients will have familiarisation on machine followed by 5 minutes rest prior to commencing arm cycling.

EXERCISE SESSION

Participants will perform arm cycling using a table-top arm cycler. Cadence and resistance of the exercise will be determined as per each individual's tolerance. All exercise will be supervised by the neuromuscular specialist physio, to ensure it is completed safely. Participants will be able to have plenty of breaks during the assessments.

The arm cycle will be placed on table with height adjusted to be the same as the acromion (highest point on shoulder). Participants will start arm cycling at a low intensity and increase as per tolerance. They will exercise for a maximum of two minutes at a time, followed by a rest period for 30 seconds. In this manner 5 cycles of exercise and rest would be performed. The exercise sessions are anticipated to last a maximum of 20 minutes, depending on the patient’s abilities.

OUTCOME MEASURES

The following measures will be assessed (and recorded in the participant CRF) prior to exercise:

•The Oxford Shoulder Score (OSS) a 12 item shoulder instability questionnaire. The OSS is a validated scoring system used to assess the degree of pain and disability caused by shoulder pathology.[14]

•The range of movements at shoulder and elbow joints will be recorded by goniometer.

- Strength of muscles at shoulder and elbow joint with portable hand held dynamometer. Muscle tested must have ability to move against gravity. “Make test” is performed where

the participant holds isometric contraction for 3-5 seconds. Dynamometer is held perpendicular to the muscle being tested at a set distance from the joint. 2-3 maximum voluntary isometric or eccentric contractions are completed and scores summed.

- Patients would have a familiarisation with arm cycling and commenting about rate of perceived exertion (RPE). This will be followed by 5 minutes rest prior to starting the exercise.

Measurements during exercise:

- Patient selected resistance and cadence (both relate to the intensity of cycling)

•Patient rating of perceived exertion ('effort') during arm cycling using the Borg Rate of Perceived Exertion (RPE) scale for each cycle of exercise.

•

•Video analysis of shoulder and elbow joint angles during arm cycling. Recording perpendicular to the plane of movement will be done. Primary view should be the sagittal view that will allow measuring wrist, elbow and shoulder flexion and extension.

Clearly visible marker will be placed on the distal head of fifth metacarpal bone, styloid process of ulna, lateral epicondyle of humerus, and lateral aspect of acromion process and most lateral part of hip on the side. That way it can be digitized and joint angles calculated by the lines between these points. The shoulder flexion extension can be relative to the person's position, or relative to the vertical. The marker on hip would help for indication of trunk orientation.

Measurements after exercise:

These are subjective questions and patients comments recorded in their verbatim:

- Patient suggestions about factors that limit their performance.
- Would they consider doing arm cycling if effectiveness is proved?
- Patients will receive a phone call 4 days later to ask "were there any consequences of the arm cycling you did 4 days ago, either positive or negative?"

SAMPLE SIZE

We plan to recruit 20 FSHD sufferers to take part in this study. This sample size is appropriate for a pilot study, intended to inform the design and sample size of a future larger study of around 60 patients. This would be the size of a study comparing between baseline and post-intervention, assuming the exercise will have an effect size (change in outcome divided by baseline SD) that can be considered "medium", and that a moderate correlation exist between baseline and post-intervention outcome (r=0.5). In that case, using a sample

size of 20 patients will minimize the total sample size for the pilot phase and the "real" clinical trial. no sample size calculations have been made here.

REFERENCES

1. Bergsma A, Murgia A, Cup E. H, Verstegen P.P, Meijer K, Groot I J. Upper Extremity Kinematics and Muscle Activation Patterns in Subjects With Facioscapulohumeral Dystrophy. Archive of Physical Medicine and Rehabilitation 2014:95(9): 1731-1741.

2. Bergsma A, Cup E.H.C., Geurts A.C.H et al. Upper extremity function and activity in facioscapulohumeral dystrophy and limb-girdle muscular dystrophies: a systematic review. Disability and Rehabilitation. 2014. E-pub 1-16

3. Attarian S, Salort-Campana E, Nguyen K, Behin A, Andoni Urtizberea J. Recommendations for the management of facioscapulohumeral muscular dystrophy in 2011. Rev Neurol (Paris). 2012 Dec;168(12):910-8.

4. Schepelmann K, Winter Y, Spottke AE, Claus D, Grothe C, Schröder R, Heuss D, Vielhaber S, Mylius V, Kiefer R, Schrank B, Oertel WH, Dodel R. Socioeconomic burden of amyotrophic lateral sclerosis, myasthenia gravis and facioscapulohumeral muscular dystrophy. J Neurol. 2010 Jan;257(1):15-23.

5. Giannini S, Faldini C, Pagkrati S, Grandi G, Digennaro V, Luciani D, Merlini L. Fixation of winged scapula in Fascioscapulohumeral Muscular Dystophy. Clinical Medicine and Research, 2007, 5: 155-162.

6. Ziaee MA, Abloghasemian M, Majd ME. Scapulothoracic arthrodesis for winged scapula due to facioscapulohumeral dystrophy. The American Journal of Orthopaedics 2006 Jul; 35(7):311-5.

7. Milner-Brown HS, Miller RG. Muscle strengthening through high-resistance weight training in patients with neuromuscular disorders. Arch Phys Med Rehabil 1988;69(1):14–9.

8. van der Kooi EL, Vogels OJ, van Asseldonk RJ, et al. Strength training and albuterol in facioscapulohumeral muscular dystrophy. Neurology 2004;63(4):702–8.

9. Olsen DB, Orngreen MC, Vissing J. Aerobic training improves exercise performance in facioscapulohumeral muscular dystrophy. Neurology 2005;64:1064–6.

10. Gianola S, Pecoraro V, Lambiase S, Gatti R, Banfi G, Moja L. Efficacy of muscle exercise in patients with muscular dystrophy: a systematic review showing a missed opportunity to improve outcomes. PLoS One. 2013 Jun 12;8(6):e65414. doi: 10.1371/journal.pone.0065414. Print 2013.

11. Voet NB, van der Kooi EL, Riphagen II, Lindeman E, van Engelen BG, Geurts AC. Strength training and aerobic exercise training for muscle disease. Cochrane Database Syst Rev. 2013 Jul 9;7:CD003907. doi: 10.1002/14651858.CD003907.pub4.

12. Jansen M, van Alfen N, Geurts A. C. H and de Groot I.J.M. Assisted Bicycle Training Delays Functional Deterioration in Boys With Duchenne Muscular Dystrophy: The

Randomized Controlled Trial "No Use Is Disuse" published online 24 July 2013 Neurorehabil Neural Repair

13. Pasotti S, Magnani B, Longa E, Giovanetti G, Rossi A, Berardinelli A, Tupler R, G. An integrated approach in a case of facioscapulohumeral dystrophy. BMC Musculoskelet Disord. 2014 May 15;15:155. doi: 10.1186/1471-2474-15-155.

14. Dawson J, Rogers K, Fitzpatrick R, Carr A. The Oxford shoulder score revisited. Arch Orthop Trauma Surg. 2009;129:119–123.
